# SIP*smart*ER delivered through rural, local health districts: adoption and implementation outcomes

**DOI:** 10.1186/s12889-019-7567-6

**Published:** 2019-09-18

**Authors:** Kathleen J. Porter, Donna Jean Brock, Paul A. Estabrooks, Katelynn M. Perzynski, Erin R. Hecht, Pamela Ray, Natalie Kruzliakova, Eleanor S. Cantrell, Jamie M. Zoellner

**Affiliations:** 1 Department of Public Health Sciences, University of Virginia, School of Medicine, 16 East Main Street, Christiansburg, VA 24073 USA; 2 College of Public Health, University of Nebraska Medical Center, 984365 Nebraska Medical Center, Omaha, NE 68198-4365 USA; 3 Virginia Department of Health, New River Health District, 212 3rd Avenue, Radford, VA 24141 USA; 4 Virginia Department of Health, Lenowisco Health District, 134 Roberts Street SW, Wise, VA 24293 USA

**Keywords:** Beverages, Behavioral research, Rural population, Implementation outcomes

## Abstract

**Background:**

SIP*smart*ER is a 6-month evidenced-based, multi-component behavioral intervention that targets sugar-sweetened beverages among adults. It consists of three in-person group classes, one teach-back call, and 11 automated phone calls. Given SIP*smart*ER’s previously demonstrated effectiveness, understanding its adoption, implementation, and potential for integration within a system that reaches health disparate communities is important to enhance its public health impact. During this pilot dissemination and implementation trial, SIP*smart*ER was delivered by trained staff from local health districts (delivery agents) in rural, Appalachian Virginia. SIP*smart*ER’s execution was supported by consultee-centered implementation strategies.

**Methods:**

In this mixed-methods process evaluation, adoption and implementation indicators of the program and its implementation strategy (e.g., fidelity, feasibility, appropriateness, acceptability) were measured using tracking logs, delivery agent surveys and interviews, and fidelity checklists. Quantitative data were analyzed with descriptive statistics. Qualitative data were inductively coded.

**Results:**

Delivery agents implemented SIP*smart*ER to the expected number of cohorts (*n* = 12), recruited 89% of cohorts, and taught 86% of expected small group classes with > 90% fidelity. The planned implementation strategies were also executed with high fidelity. Delivery agents completing the two-day training, pre-lesson meetings, fidelity checklists, and post-lesson meetings at rates of 86, 75, 100, and 100%, respectively. Additionally, delivery agents completed 5% (*n* = 3 of 66) and 10% (*n* = 6 of 59) of teach-back and missed class calls, respectively. On survey items using 6-point scales, delivery agents reported, on average, higher feasibility, appropriateness, and acceptability related to delivering the group classes (range 4.3 to 5.6) than executing missed class and teach-back calls (range 2.6 to 4.6). They also, on average, found the implementation strategy activities to be helpful (range 4.9 to 6.0). Delivery agents identified strengths and weakness related to recruitment, lesson delivery, call completion, and the implementation strategy.

**Conclusions:**

In-person classes and the consultee-centered implementation strategies were viewed as acceptable, appropriate, and feasible and were executed with high fidelity. However, implementation outcomes for teach-back and missed class calls and recruitment were not as strong. Findings will inform the future full-scale dissemination and implementation of SIP*smart*ER, as well as other evidence-based interventions, into rural health districts as a means to improve population health.

**Electronic supplementary material:**

The online version of this article (10.1186/s12889-019-7567-6) contains supplementary material, which is available to authorized users.

## Background

Understanding the implementation of effective interventions is critical to promoting their sustained translation into practice-based settings and enhancing their potential impacts on population health. Successful implementation impacts both service and client outcomes [1, 2]. In combination with understanding the generalizability of an intervention’s reach into the intended population and its adoption and maintenance at the organizational level, implementation is key factor in replicating effective interventions in typical community or clinical settings [1]. Further, additional implementation outcomes including acceptability, appropriateness, costs, and feasibility can inform the external validity of evidence-based interventions when delivered outside of the research context [2].

SIP*smart*ER is a six-month, multi-component, community-based, behavioral intervention designed to reduce the intake of sugar-sweetened beverages (SSBs) among rural, Appalachian adults [3, 4]. SSBs are non-alcoholic beverages that contain sugar and few other nutrients, such as soda/pop, energy drinks, sweet tea, and fruit drinks. SIP*smart*ER is grounded in the Theory of Planned Behavior [5] and health literacy principles [6]. The intervention design is described in more detail elsewhere [3]. It is one of only two known interventions targeting SSB intake among adults that have demonstrated significant improvement in adults’ SSB consumption [3, 4, 7–9]. SIP*smart*ER also is the only SSB-focused intervention included in the National Cancer Institute’s repository of Research-Tested Intervention Programs (RTIPs) [10]. Translating SIP*smart*ER into practice-based settings is important given the numerous preventable health conditions associated with excessive SSB intake (e.g., obesity, diabetes, heart disease, cancer, dental caries) [11–15], particularly among populations with low socio-economic status and/or living in rural areas [16–19]. Given SIP*smart*ER’s demonstrated effectiveness [4], it is important to explore how it could be disseminated, implemented, and integrated within a system that reaches health disparate communities in Appalachia.

A pilot dissemination and implementation (D&I) trial was collaboratively developed with medical directors and leadership staff from the four local health districts within the Virginia Department of Health (VDH) that service the rural Appalachian counties [20]. The trial design was grounded in the RE-AIM framework [1] and the Interactive Systems Framework [21, 22], and the evaluation was guided by RE-AIM. Specifically, this pilot trial was designed to measure the reach, effectiveness, adoption, and implementation of SIPsmartER when delivered through local health districts. To support the delivery of SIP*smart*ER, the research team and health department stakeholders developed and applied an implementation strategy [23] that would build both the general and innovation specific capacity necessary to deliver SIP*smart*ER. This strategy utilized the key elements of consultation identified by Edmunds (e.g., on-going instruction, self-evaluation, and feedback) [24].

The purpose of this paper is to describe SIP*smart*ER’s adoption and implementation when delivered through rural, local health districts [1, 2]. In addition, determinants of adoption, implementation, and organizational maintenance that align with the Interactive Systems Framework were assessed from the perspective of the delivery agents from the local health districts: (i) acceptability (satisfaction with aspects of the innovation), (ii) appropriateness (perceived fit, relevance, and suitability), and (iii) feasibility (actual fit). This paper specifically focuses on outcomes related to both delivery expectations and the implementation strategy.

## Methods

This study is a mixed-methods process evaluation of a pilot type 2 hybrid effectiveness-implementation trial of SIP*smart*ER [20]. A type 2 hybrid effectiveness-implementation trial allows for the simultaneous testing of an intervention and an implementation strategy to supports its delivery [20]. It specifically reports on SIP*smart*ER’s adoption and implementation at the organizational-level. A complete description of effectiveness outcomes are outside the scope of this manuscript. However, during this trial, significant improvements from baseline to 6-months in SSB intake (− 403(CI = − 528, − 278) kcals/day, (*p* < 0.001)), which were comparable to findings from the effectiveness trial [4]. Also, significant changes (all *p* < 0.05) in SSB-related attitudes, perceived behavioral control, behavioral intentions, and media literacy were observed.

Study procedures were approved by the Institutional Review Boards of Virginia Tech, the University of Virginia, and Virginia Department of Health. Written informed consent was obtained from health department staff.

### SIP*smart*ER type 2 hybrid effectiveness-implementation trial

#### Identification and logistics of health districts and delivery agents

Each of the four southwest Virginia health districts invited to participate in this trial agreed to participate. These four districts serve the same counties and cities as the effectiveness trial [3]. These areas consistently score among the poorest across Virginia on the Health Opportunity Index [25], are federally designated as medically-underserved [26] and have an average rurality status of 6/9 [27].

Medical directors were asked to identify the staff within their district who would be ideal delivery agents to implement SIP*smart*ER. The number of SIP*smart*ER cohorts each district agreed to deliver was determined by budget and power calculations, with each health district expected to deliver two to four cohorts. Budgets were planned collaboratively with medical directors during the grant writing process. Each health district was provided with a sub-contract reflecting the expected percent effort necessary for delivery agents to implement SIP*smart*ER.

Following awarding of the grant, planning meetings were held between research staff and VDH medical directors and delivery agents to help ensure the compatibility of the approach with the health department delivery system [22, 28] and to devise a plan for the division of SIP*smart*ER implementation and research tasks between VDH delivery agents and research staff. This plan, detailed in Table 1, was adjusted to better reflect the needs of the delivery agents following the in-person training, the first implementation strategy activity. Notably, the extent of the role of the delivery agents in (i) the completion of teach-back and missed class calls and (ii) participant engagement activities was reduced. During one of these meetings, delivery agents created specific action plans for recruitment of participants within their districts.

**Table 1:** Distribution of SIP*smart*ER delivery tasks between Virginia Department of Health (VDH) staff and the research team

	Plan by researchers & medical directors	Refined plan by delivery agents
Cohort Recruitment		
Administer screening surveys	VDH	VDH-led; Research team to help as needed
Call and schedule participants for baseline enrollment	Research team	Research team
Intervention Delivery		
Teach 3 SIP*smart*ER lessons	VDH	VDH
Conduct missed class calls	Researchers & VDH split	Researcher-led; VDH to assist after 1st cohort
Conduct teach-back calls	Researchers & VDH split	Researcher-led; VDH to assist after 1st cohort
Reminder calls	Researchers & VDH split	Research team
Reengagement calls	Research team	Research team
Track completion/attendance	Researchers & VDH split	Research team
Manage IVR system	Research team	Research team
Research Outcome Assessment		
Conduct health assessments	Research team	Research team
Appointment postcards	Research team	Research team

#### SIP*smart*ER intervention components

SIP*smart*ER consists of three lessons delivered through group classes, one teach-back call, and eleven interactive voice response (IVR) calls. Participants who did not attend classes had the opportunity to complete the lesson as a missed class phone call. In the classes, participants received instruction on core content necessary to increase motivation and skills to decrease SSBs. During the teach-back call, participant to review content from the first class and complete a personal action plan with a trained research assistant. Through the IVR calls, participants identified their ounces of SSB intake, completed an action plan, and received a motivational message. Intervention activities and materials have been described in detail elsewhere [3, 10, 29–31]. In addition to intervention components, there were specific activities to support participant retention, including re-engagement calls to participants who had not completed two activities in a row [32].

#### SIP*smart*ER delivery timeline

An initial staggered plan of implementation per district was planned with districts starting SIP*smart*ER cohorts at different times. A goal of 10 or more participants per cohort was set. However, during the trial, districts completed cohorts at similar times. First cohorts were completed between Fall 2016 and Spring 2017 (*n* = 6) with additional cohorts completed between Summer 2017 and Spring 2018 (*n* = 6).

#### Consultee-centered implementation strategy

An implementation strategy that utilized a consultee-centered approach was drafted by the research team based on the principles outlined by Edmunds and colleagues. This approach involves non-hierarchical interactions between a consultant (e.g., researcher) and consultee (e.g., delivery agent) through which the consultant provides guidance to the consultee related to a current work problem that is within the scope of the consultant’s expertise [24, 33]. Through these interactions, consultees master general skills and build specific skills related to problem-solving implementation barriers and appropriately adapting intervention components. They are also held accountable for delivering the evidence-based program [33].

Then, to ensure the training was compatible with the health department processes for adopting new interventions, the plan for the implementation strategy was presented to the medical directors and delivery agents for feedback and changes were made based on their feedback. Final implementation strategies addressed three of the four consultation techniques identified in Edmund’s review: on-going instruction, self-evaluation, and feedback [24]. The expectations for engagement in each of these activities varied by timing of cohort, as delivery agents were expected to complete more implementation strategy activities during their first cohort(s) than subsequent ones.

#### On-going instruction

Five implementation strategies related to on-going instruction were utilized.

##### Recruitment how-to handout

Materials were created for delivery agents and other VDH staff to support participant screening for enrollment. A handout provided scripting and frequently asked questions to engage potential participants. This strategy was seen as more compatible with medical directors’ expectations for recruitment strategies and, as such, provided a relative advantage [22, 28] over other proposed activities, including in-person or teleconference training.

##### Two-day training

A two-day (~ 12 h), in-person training was held in August 2016, prior to the start of the first cohorts. Two researchers facilitated the training. The training utilized several educational strategies: didactic presentation of foundational principles and key content areas of lessons, modeling lesson activities and calls, and interactive discussions to identify potential barriers and possible solutions. During this training, delivery, agents received instruction in the core principles for SIP*smart*ER, a comprehensive review of the SIP*smart*ER intervention components, and practical tips for delivering lessons and calls. Additionally, they practiced delivering lesson activities. Delivery agents also received lesson manuals that included (i) lesson plans with lesson objectives, procedural steps for delivering lessons, background information on key lesson content, and supply lists, (ii) PowerPoint slides to support the execution of the lesson activities, and (iii) participant handouts. These activities aligned with reducing the complexity of the intervention by providing all training and participant-facing materials in a package that could be easily accessed by the delivery agents [22, 28].

##### Pre-lesson meetings

During the delivery of their first cohort, delivery agents had short (~ 20 min meetings) before each lesson with one of the researchers who had delivered SIP*smart*ER during the effectiveness trial. These conversations provided time for the delivery agents to have questions and concerns about the lesson addressed. Also, the researcher provided tips for executing lesson activities based on past experiences delivering the lessons. These meetings were optional after a delivery agent’s first cohort.

##### Co-teaching

Delivery agents had the option to co-teach each lesson during their first cohort with one of the researchers. If co-teaching was requested, the delivery agent and the researcher would discuss logistics for the co-teaching during the pre-lesson meeting.

##### Lesson recap videos

Lesson overview videos were developed for delivery agents to watch prior to delivering their second cohorts, which were approximately 10 months after the in-person training. These videos reviewed the foundations and design of the curriculum, content background, and specific lesson flow and activities.

#### Self-evaluation and feedback

Delivery agents completed a fidelity checklist immediately following the delivery of each lesson and were provided with feedback through lesson observations and post-lesson meetings. A member of the research team observed each delivery agent deliver SIP*smart*ER the first two times they taught each lesson. During the observations, the researcher completed a fidelity sheet and took field notes. Following observed lessons, the delivery agent(s) and the researcher had a short (< 10 min) audio-taped discussion about the lesson delivery, including highlights of the lesson and areas for improvement. Additional instruction was provided if aspects of the lesson delivery (e.g., execution of activities, inclusion of improper content) needed improvement.

### Measures

To assess engagement in and perceptions of implementation strategy activities and actual implementation, data from eight measures were used: (i) cohort recruitment logs, (ii) delivery agent engagement logs, (iii) post-training surveys, (iv) post-training interviews, (v) fidelity checklists, (vi) post-cohort interviews, (vii) post-cohort surveys, and (viii) capacity surveys. These measures allowed for a concurrent mixed methods assessment of SIP*smart*ER’s implementation and adoption.

#### Cohort recruitment logs

Logs of health district recruitment activities were maintained as a means of tracking the number of cohorts each district recruited.

#### Delivery agent engagement log

Logs were maintained to track delivery agents’ fidelity to delivery expectations and implementation strategy activities.

#### Post-training surveys and interviews

Post-training surveys and interviews were completed after the 2 day in-person training and before each delivery agents’ first cohort. Interviews were audio-recorded. These measures captured information from the delivery agents related to the appropriateness of SIP*smart*ER and its components within the health district and the delivery agents’ regular job functions. Survey items included question about delivery agents’ confidence to complete delivery expectations and implementation strategies and their perceived feasibility of doing so. Items were measured using 6-point Likert scales. Post-training interviews also assessed the adoption and feasibility of program recruitment. Please see Additional file 1 for these instruments.

#### Fidelity checklists

Unique fidelity checklists were developed for each of the three lessons. These checklists assessed the degree to which a specific lesson’s activities were completed (none = 0, partial = .5, all = 1) and if the activity was modified (no = 0, yes = 1). There were also sections to enter specific notes about the implementation. These checklists were completed by delivery agents after each delivered lesson and by a researcher after each observed lesson. Please see Additional file 2 for these instruments.

#### Post-cohort surveys and interviews

Post-cohort surveys and interviews were completed after each delivery agent completed a round of cohorts. Interviews were conducted by a researcher who had limited involvement with delivery agents during intervention delivery activities. These measures captured information from the delivery agents related to the feasibility and acceptability of SIP*smart*ER, its components, and the implementation strategy. Post-cohort surveys also assessed the fidelity to delivery expectations. Scaled items on the post-cohort survey were measured using 4-point Likert scales. Please see Additional file 3 for these instruments.

#### Capacity surveys

After completing all their cohorts, delivery agents were asked to complete a survey with open-ended questions. Questions were related to the acceptability and appropriateness of maintaining SIP*smart*ER in their health district, including the resources they would need to sustain the program. Please see Additional file 4 for this instrument.

### Analysis

A concurrent mixed-methods approach was used to analyze data [34]. Data from each measure were analyzed independently using the methods described below. Then, qualitative and quantitative data measuring the same adoption or implementation indicators were converged to allow findings to be compared across measures.

#### Quantitative analyses

Frequencies of completing delivery expectations and engaging in implementation strategy activities were calculated. Means and standard deviations were calculated for items on post-training surveys, post-cohort surveys, and fidelity checklists and are presented by district and overall. To make results from post-training and post-cohort surveys more comparable, post-cohort survey scores were transformed from 4-point Likert scale scores to 6-point Likert scores using linear stretch [35]. An average fidelity score and average activity modification for each delivered lesson was calculated by averaging the fidelity ratings and modification ratings for each lesson activity.

#### Qualitative analyses

Transcripts of post training and cohort interviews and open-ended questions from capacity surveys were coded using a constant comparative approach by two researchers [36]. Transcripts and open-ended responses on capacity surveys were first organized into categories that reflected the major delivery expectations (recruitment, lesson delivery, and teach-back and missed class calls), implementation strategy, and sustainability. Content within these categories were reviewed for emerging themes. These themes were reviewed and organized to create codes that were applied to the categories. One researcher applied the codes to the transcripts and surveys while another reviewed coding to ensure the appropriate text was captured. Researchers discussed and resolved any differences. This process was repeated within codes to identify more discrete units as needed [37].

## Results

### Adoption and recruitment

Table 2 presents data related to adoption of SIPsmartER by rural, local health departments, a description of delivery agent roles within each district, and medical director turnover during the implementation process. Delivery agents led or participated in the recruitment of 89% (*n* = 17) of cohorts. More cohorts were recruited for than were enrolled (19 recruited, 12 enrolled). Two health districts met their target number of enrolled cohorts while one district enrolled one less and another enrolled one more.

**Table 2:** Rural, Local Health Districts Adoption of and Recruitment for SIP*smart*ER

	Overall	District 1	District 2	District 3	District 4
Delivery Agents					
Number of participating delivery agents	7	1	2	3	1
Educator roles outside of SIPsmartER delivery	Health educator (*n* = 4) Health educator/ WIC nutritionist (*n* = 1) WIC nutritionist (*n* = 1) Public health nurse (*n* = 1)	Health educator/ WIC nutritionist (*n* = 1)	Health educator (*n* = 2)	Health educator (*n* = 1), WIC nutritionist (*n* = 1), Public health nurse (*n* = 1)	Health educator (*n* = 1)
Medical Directors					
Districts with medical director changes during implementation	3 (75%)	yes^a,b^	no^b^	yes^a^	yes
Timing of medical director change	–	Initial left: Mid-way through 1st cohort New started: Mid-way through 1st cohort	n/a	Initial left: Mid-way through 1st cohort New started: By end of 1st cohort	Initial left: Mid-way through 1st cohort New started: After end of 1st cohort
Recruitment					
Number of cohorts expected	12	2	4	2	4
Number of cohorts recruited	19 (173%)	2 (100%)	7 (175%)	3 (150%)	5 (125%)
Number of cohorts recruited by delivery agent	17/19 (89%)	2/2 (100%)	7/7 (100%)	3/3 (100%)	3/5 (60%)
Number of cohorts enrolled	12 (100%)	2 (100%)	4 (100%)	3 (150%)	3 (75%)

^a^ Shared the same medical director at start of program ^b^ Shared the same medical director at the end of the program

### Implementation Fidelity

Implementation fidelity data are presented in Table 3.

**Table 3:** Delivery Agent Fidelity to Intervention Delivery and Implementation Strategy Activities

	Overall	District 1	District 2	District 3	District 4
Intervention Delivery					
*Group Classes*					
Number of classes expected based on enrolled cohorts	36	6	12	9	9
Classes delivered	31/36 (86%)	5/6 (83%)	9/12 (75%)	7/9 (78%)	10/9 (111%)
Division of teaching tasks between delivery agents	Varied by district	The 1 identified delivery agent taught all classes	The 2 identified agents co-taught all classes	▪ Delivery agent #1: solo taught 3 classes and co-taught 2 classes with Delivery Agent #2 ▪ Delivery agent #2: taught 1 class and co-taught 2 classes with Delivery Agent #2 ▪ Delivery agent #3: taught 1 class ▪ Researcher taught 1 class ▪ Two cohorts had a combined class	The 1 identified delivery agent taught all classes
Researcher observed lesson fildelity ^a^*	93%	97%	92%	87%	96%
Researcher observed modifications^b^	17%	19%	18%	14%	19%
*Teach-Back and Missed Class Calls*					
Number of delivery agents attempting teach-back calls	3/7 (43%)	1/1 (100%)	1/2 (50%)	0/3 (0%)	1/1 (100%)
Number of teach-back calls successfully completed by delivery agents (compared to research staff)	3 (of 66, 5%)	0 (of 10, 0%)	1 (of 17, 6%)	0 (of 28, 0%)	2 (of 18, 2%)
Number of delivery agents attempting missed class calls	5/7 (71%)	1/1 (100%)	1/2 (50%)	2/3 (67%)	1/1 (100%)
Number of missed class calls successfully completed by delivery agents (compared to research staff)	6 (of 59, 10%)	0 (of 10, 0%)	0 (of 15, 0%)	6 (of 22, 27%)	0 (of 11, 0%)
Implementation Strategy					
Delivery agents completing 2 day training	6/7 (86%)	1/1 (100%)	2/2 (100%)	2/3 (67%)	1/1 (100%)
Expected pre-meetings completed^c^	9/12 (75%)	1/3 (33%)	3/3 (100%)	3/4 (75%)	3/3 (100%)
Additional pre-meetings completed	9	3	3	–	3
Classes co-delivered with researcher	3/32 (10%)	2/5 (40%)	0/0 (0%)	1/8 (13%)	0/10 (0%)
Fidelity check lists completed by delivery agent for each class taught	43/43 (100%)	5/5 (100%)	Delivery Agent #1 = 9/9 (100%) Delivery Agent #2 = 9/9 (100%)	Delivery Agent #1 = 6/6 (100%) Delivery Agent #2 = 3/3 (100%) Delivery Agent #3 = 1/1 (100%)	10/10 (100%)
Delivered classes observed by researcher	27/32 (84%)	5/5 (100%)	6/9 (67%)	8/8 (100%)	8/10 (80%)
Post-meetings completed^c^	27/27 (100%)	5/5 (100%)	6/6 (100%)	8/8 (100%)	8/8 (100%)

^a^ Score for each lesson is an average of the fidelity ratings or modifications from all the classes observed by the researcher for that lesson

^b^ Score for each lesson is an average of the fidelity ratings or modifications from all the classes reported by delivery agents for that lesson

^c^ Number of expected pre- and post-meetings varied vary based on division of teaching responsibilities and co-teaching. Pre-meetings were expected to be completed prior to the first time each educator (or pair of educators) delivered a lesson. Post-meetings were expected after each completed and observed class

* difference between research observed and delivery agent reported fidelity *p* < 0.01

#### Intervention delivery

##### Lesson delivery

Of the anticipated 36 classes (one class for each of the three lessons planned per implemented cohort), 31 (86%) were delivered by delivery agents and one by a researcher. For the two health districts with multiple delivery agents, distribution of teaching responsibilities were approached differently, with one delivery agents in one district co-teaching all lessons and the district’s delivery agent alternating classes.

An average of 93% fidelity was found across all lessons and districts based on fidelity checklists completed during researcher observations. Based on researcher observation, delivery agents modified 17% of activities. Most of these modifications were appropriate, including tailoring examples to be more locally relevant (e.g., adding pictures of popular regional sugary drinks), making activities more suitable for audience (e.g., adapting activities using worksheets so they better met the needs of a very low literate group), and adding extra content to make a concept clearer (e.g., adding a parody of a famous sugary ad created by a health watch group into a lesson addressing advertising) [38]. However, one modification was not appropriate: the addition of health risks that are not well-supported by scientific literature.

##### Teach-back and missed class calls

Three delivery agents (43%) attempted teach-back calls and five delivery agents (71%) attempted missed class calls. Overall, agents completed three (of 66, 5%) teach-back calls and six (of 59, 10%) missed class calls.

The actual process for completing the teach-back and missed class calls evolved from the plan devised following the training. To maximize both call completion and agent involvement in calls, delivery agents were not assigned specific participants for the calls. Instead, during call periods, delivery agents provided times they could make calls within their work schedule. Then, the research staff would provide delivery agents with a list of participants to call based on whether participants preferred call times were within the delivery agent’s window of availability. Researchers made the calls when the delivery agents were not available.

#### Implementation strategy

Six (86%) delivery agents attended both days of the in-person training; the other attended only 1 day (due to a family emergency). Eighteen pre-meetings were held: nine of the expected 12 (75%) pre-meetings that were held before each delivery agent’s first time teaching a lesson and nine additional during the delivery agents’ second time teaching the lessons. Three (10%) of the delivered classes were co-taught. Each delivery agent completed a fidelity checklist every time after s/he taught a class for a total of 43 checklists (100%). Twenty-seven classes (84%) were observed by researchers. The five classes that were not observed were the third time delivery agents taught the lessons, so they did not require an observation per the protocol. Post-lesson meetings were completed after all observed lessons.

### Acceptability, appropriateness, and feasibility

Quantitative and qualitative findings about implementation acceptability, appropriateness, and feasibility are presented in Tables 4 and 5, respectively.

**Table 4:** Delivery Agent Quantitative Assessment of Delivery Expectations and Implementation Strategy

Item		Post-Training (*n* = 7)	After 1st round of delivery (*n* = 6)^a^	After 2nd round of delivery (*n* = 4)^b^
Intervention delivery		Mean (SD)	Mean (SD)	Mean (SD)
Lesson Delivery	Confidence to adequately prepare for classes	5.0 (0.6)		
	Perceived feasibility to adequately prepare for classes	4.3 (1.0)		
	Perception of how well they adequately prepared for classes^d^		5.2 (0.9)	5.6 (0.8)
	Confidence to meet lesson objectives when delivering^c^	5.0 (0.6)		
	Perception of if they met lesson objectives when delivering^c,d^		5.2 (0.8)	5.4 (0.8)
	Confidence to meet the learning needs of participants	4.8 (0.8)		
	Perception of if they met the learning needs of participants^d^		5.4 (0.9)	5.6 (0.8)
Participant management	Perceived feasibility to call participants about classes	3.1 (1.3)		
	Perceived feasibility to track participant attendance	4.1 (1.3)		
Teach-back calls	Confidence to deliver the teach-back call	4.6 (1.3)		
	Perceived feasibility to deliver the teach-back call	2.6 (1.5)		
Missed class calls	Confidence to deliver missed class calls	4.6 (1.3)		
	Perceived feasibility to deliver missed class calls	2.4 (1.3)		
Implementation Strategy				
General	Confidence that will get the necessary support from SIP*smart*ER staff	4.9 (1.2)		
	Perception of how well they received necessary support from SIP*smart*ER staff^d^		5.7 (0.7)	6.0 (0.0)
Two-day training	Satisfaction with training length	5.3 (0.5)		
	Satisfaction with material presentation	5.3 (0.8)		
	Perceived helpfulness of the 2 day training^d^		5.4 (0.9)	6.0 (0.0)
Pre-lesson meetings	Perceived helpfulness of pre-lesson meetings^d^		5.4 (0.9)	6.0 (0.0)
Fidelity checklists	Perceived feasibility to complete fidelity check-lists	4.7 (1.0)		
	Perceived helpfulness of fidelity checklists		4.9 (1.3)	5.6 (0.8)
Lesson observations	Perceived helpfulness of lesson observations		5.4 (0.9)	6.0 (0.0)
Post-lesson meetings	Perceived feasibility to complete lesson debriefings	4.1 (1.1)		
	Perceived helpfulness of post-lesson meetings^d^		5.2 (1.4)	6.0 (0.0)

^a^ *n* = 6 because one delivery agent did not complete the post-cohort survey after the first round of delivery (Fall 2016-Spring 2017)

^b^ *n* = 4 because only four delivery agents delivered SIP*smart*ER during the second round as one of the districts completed their cohorts during the first round of delivery (Summer 2017 – Winter 2018)

^c^ Score is an average of delivery agent rating for individual items for each of the three lessons

^d^ Items were on a 4-point agreement or helpfulness scale that was rescaled to a 6-point scale

**Table 5:** Delivery Agent Qualitative Assessment of Recruitment, Delivery Expectations, Implementation Strategy

	Positive	Negative	Example Quotes^a^
Recruitment			
Recruitment	• Targeting intact groups instead of more broadly recruiting in the community • Having recruitment materials and adjusting them to reflect needs of population	• Perceived lack of interest by community • Difficult to engage other staff in recruitment	+ “I think finding those groups that are already together has been key for us. I think we would still be struggling if we were just reaching for general community members” +“For us, the only way it started to become successful was when we had our brochure to give to people early on” -“…we’re hitting the people we need to hit; it’s just been trying to find a way to get them interested in realizing ‘OK, yes, I’m consuming too many sugary drinks and maybe need to stop’ and then get them interested in taking this class” - “…[what] was said that we were going to do, each employee was going to do 4 surveys. And, for I don’t know how that approach is working for other districts, but for us, it’s just not feasible to ask other employees who already have a busy schedule”
Intervention Delivery			
Lesson Delivery	• Program was well developed • Aligned with professional training/experience of educators • Able to modify lessons to meet needs of population • Participant interactions with one another • Seeing participants make changes	• Class attendance issues	+ “Well I think the classes were designed very well. You know each on kind of followed a natural, kind of logical progression and slow” + “I’m a health educator so this is what I do. I teach a lot and I have that ability and I know how to read my audience to get information” + “We just had a great group that was very supportive of each other and very interactive” + “We found a couple of things that ‘hey this is what people here drink so let’s get it in here too’ and then we added a couple [of images] to the celebrity [marketing slide]. We added a couple that we felt like folks would relate to me than what already existed so you know, we just tweaked it here and there, but it was very helpful that we were allowed to do that because it helped with our presenting… It gave us flexibility without losing you know the content” +“I feel like the most positive thing was actually seeing the change because I think that the group from [location 1], it was tremendous though the outcome from where they started to the end” + “Well, I feel like the most positive thing was actually seeing the change because I think that the group from [location 1], it was tremendous though the outcome from where they started to the end” - “I guess the biggest thing would be getting people to come back and keeping them interested throughout the program, and of course, you have that with any program” - “The only thing for me is just not having enough people actually sitting in the classroom and that’s really hard when you have an audience of two to get a lot of interaction”
Teach-Back & Missed Class Calls	• Calls provided a nice way to connect with participants about content, either reviewing or missed	• Perceived as not feasible because of time commitment • Scheduling can be difficult if participants need to be contacted outside of normal business hours	+ “I found it very useful for me to see what they retained and then knowledge wise and then making the connection and just saying good luck” + “Yeah, the missed class calls are good because that brings people up to speed and also lets them know ‘hey you missed it but you know we’re gonna bring [you] up to date’” - “For me it is completely unfeasible. I mean with my time and what I cover on the day-to-day basis, I personally could not do it” - “It’s just gonna be hard to get those in with you know the regular day. You never have enough time anyway” - “You know most people want call backs at night and that doesn’t really fit with most work schedules”
Implementation Strategy			
General	• Research staff had content expertise • Able to effectively communicate with research staff about needs and questions	• Support did not seem necessary as content was not new	+“It was helpful to have you guys there during the class so if there was any question I could not answer or address… you were able to answer that question” +“It allowed us to have honest and open communication and I think that was the most important thing” −/+“I mean, initially, I was a little uncomfortable, I thought you know, this information is not anything new to me and why does somebody have to observe me doing it … but after the first lesson I didn’t feel like anybody was there to judge me but really to judge the program itself”
Training Specific	• Provided a complete picture of the program • Set expectations clear expectations for what was required • Opportunity to develop a network with researchers and other delivery agents		+ “It was really good to see the lessons as well as the calls kind of modeled for you just to get a general idea of, you know, the flow of the class, the timing and things like that. I think that that was really the strong point” +“So that was huge for use to actually be able to go through the mock sessions and to understand what our audience might be thinking and then be able to ask the right questions so that we all got the best answers.”
General Comments			
Need	• Addresses a behavioral and health needs in the community • Extends existing programming	• Duplicates other (less intense) programming	+ “I think it will be really good for them, especially in our area we have a culture of Mountain Dew drinking and things like that so I think shining a light on sugar-sweetened beverages will have a really big impact” + “…I think it’s something that’s incredibly useful … there’s of course Rev. Your Bev and like different events or different campaigns that are out here as far as drinking more water but not like actually having participants set goals, giving them strategies and things like that” -“We you know, already teach a similar curriculum”
Logistics		• Length of program • Focus of staff is dictated by funds	- “Over that you know fourteen plus week timeframe, it’s a large commitment for anybody” - “Our priorities are determined by the funds that are coming into the health department”

^a^ + = positive statement and - = negative statement

#### Recruitment

Delivery agents reported that recruitment was often difficult, noting the community seemed uninterested in the program and it was hard to engage other health department staff to support recruitment efforts. They also identified successful approaches to recruitment, including targeting intact groups and having handouts they could use to describe the program during recruitment.

#### Intervention delivery

##### Lesson delivery

After the training, delivery agents reported high self-ratings of confidence in preparing for lesson delivery (5.0/6), meeting lesson objectives when teaching (5.0/6), and meeting the learning needs of participants (4.8/6). They also reported moderate feasibility in their ability to adequately prepare for classes (4.3/6). After delivering SIPsmartER, delivery agents perceived their actual delivery performance to be positive, with average scores ranging from 5.2/6 to 5.6/6. Related to participant retention activities, they perceived their ability to complete reminder calls to participants about classes as slightly unfeasible (3.1/6) and their ability to track participant attendance at classes as slightly feasible (4.1/6).

From interviews and capacity-surveys, delivery agents noted low class attendance was an issue for some cohorts. However, they also reported positive aspects of delivery, including observing participants change over time, interactions among participants, and their conversations with participants. They also expressed liking the flexibility to make lesson adjustments to better meet the needs of the population and that the program was well developed and the group lessons were within their scope of professional practice.

##### Teach-back and missed class calls

On the post-training survey, delivery agents reported moderate to high confidence in their ability to complete teach-back (4.6/6) and missed class calls (4.6/6). However, ratings of the feasibility of conducting these calls were moderately unconfident (2.6/6 and 2.4/6, respectively).

Delivery agents reported seeing the utility of the teach-back calls as a way to connect with participants one-on-one and to ensure they understood the information. However, they perceived both the teach-back and missed class calls as time consuming and potentially an incompatible with their normal schedules.

#### Implementation strategy

Overall, delivery agents felt confident they would receive necessary support from SIP*smart*ER research staff (4.9/6) and reported they felt highly satisfied that they received the support they needed (5.7/6). Delivery agents reported they liked that the research staff were knowledgeable and were able to communicate effectively about general needs and questions. One delivery agent expressed initial concern that the level of support, particularly researcher observations, felt initially like their purpose was to judge the delivery agents; however, after engaging in the implementation strategies realized that they were to designed to improve the delivery of the program.

##### In-person training

Delivery agents were moderately to completely satisfied with the length of (5.3/6) and presentation at (5.3/6) the in-person training. After completing their first cohort, they rated the training as very helpful (5.4/6). Delivery agents commented they liked that the training provided a complete picture of the program, set expectations for roles, and allowed them to develop a network with the research team and peers in other health districts.

##### Other strategies

Following training, delivery agents reported that completing fidelity checklists (4.7/6) and post-lesson meetings (4.1/6) would be slightly to moderately feasible. Following completion of their first and second rounds of cohorts, delivery agents reported that the implementation strategies were helpful to very helpful, with a range of ratings from 4.9/6 to 6.0/6.

#### General impressions of acceptability, appropriateness, and feasibility

Through the interviews and capacity surveys, delivery agents from three of the four districts felt SIP*smart*ER filled a gap in their programming that targeted an important health need. However, agents from the other district felt that SIP*smart*ER was not different from already established efforts in their district that targeted the same health behavior. Delivery agents also expressed concerns about some of the program logistics, particularly the length of the program. Delivery agents across all districts reported concerns about funding, as many of the health education efforts of health department employees, especially health educators, are driven by grants.

## Discussion

This hybrid effectiveness-implementation trial examined adoption and implementation factors related to SIP*smart*ER, an evidence-based intervention to reduce sugary beverage consumption in a region experiencing health disparities. Through the use of a multi-component implementation strategy that included a consultee-centered approach, it was demonstrated that SIP*smart*ER could be adopted and implemented with high fidelity across four rural public health districts and by delivery agents with different roles within the health districts. This study contributes to the ISF literature in that findings demonstrate that the packaging an evidence-based intervention to reduce complexity and use of a facilitation process including consultee-centered training can result in the adoption and high-fidelity implementation of an intervention to address sugary beverage consumption in underserved communities. However, findings also found that the potential for sustainability and broader adoption could be jeopardized by intervention features that were less feasible and required research facilitation due to a lack of compatibility with the health department context and a lack of perceived relative advantage. Specifically, delivery agent-initiated telephone contacts were difficult to consistently implement while the in-person small group sessions were more compatible with health department practices. Similarly, qualitative feedback indicated that agent delivered telephone calls and the recruitment processes may not be as feasible.

While public health systems are beginning to play an increasing role in the implementation of evidenced-based interventions targeting chronic health conditions, little is known about how evidence-based programs can be best implemented and sustained in these systems [39–42]. Related to recruitment, fidelity to the protocol was high in terms of delivery agents being able to recruit 89% of the cohorts. However, findings from post-training and post-cohort interviews indicate low perceptions of feasibility, with most delivery agents clearly expressing their frustration with the recruitment process. Delivery agents employed two broad strategies to recruit participants: (1) surveying of the community through canvassing health department customers or the general community through local events and (2) targeting established groups, including housing and work sites [43]. The former was the strategy most districts started with and it was much less efficient. Recruiting for adult participants within the community for a program consisting of group classes and having to reach a threshold of approximately ten participants to start a class was not a common practice for any of the districts. The lack of experience and protocols within the districts may have weakened the potential reach of the program. This finding highlights the potential usefulness of systematically collecting and recording key patient health behaviors as a means to efficiently identify patients who would be good candidates for interventions [44]. It also highlights the need to consider efficient recruitment strategies that could vary based upon community resources [32].

Delivery agents’ engagement with and perceptions of the consultee-centered implementation strategies demonstrated high fidelity to these activities and reported high perceptions of acceptability, appropriateness, and feasibility. This finding is notable as it provides support for the use of consultation as an implementation strategy in community-based interventions when using professional health district staff as delivery agents. Although consultation is regularly used as an implementation strategy and has potential for use across contexts [45], its use is most commonly reported within the context of supporting community and clinic-based mental health professionals to implement evidence-based programs [33]. The strong implementation fidelity may be due to the design of the consultee-centered approach [24]. Particularly, allowing for a non-hierarchical relationship between the researchers (consultant) and delivery agent (consultee) acknowledged the past training and professional experiences of the delivery agents. Also, being able to adjust the intensity of instruction and feedback activities to reflect the growing skill and content mastery of the delivery agents with the intervention allowed them to still feel well-supported without overburdening them. However, it is important to note, that clear explanations of the purpose of a consultation approach and the specific reasons activities were chosen is needed, as the relative intensity of the strategy made one delivery agent uncomfortable initially. She felt like she was being judged and that the level of support was not needed; yet, after engaging with the implementation strategy, she recognized its purpose was not to judge her but to support the program delivery.

Our findings about the implementation of SIP*smart*ER in these rural, local health districts reflect previously identified benefits, facilitators, and barriers of implementing evidence-based programs in public health agencies. Related to benefits, findings suggest that by implementing SIP*smart*ER, their districts added programming that better addressed common risk factors for disease [46]. Delivery agents demonstrated contrasting views of some agents on the relative advantage of the approach from three districts. Three districts mentioned SIP*smart*ER was similar to another program they implement (a statewide intervention to reduce sugar-sweetened beverages that includes social marketing and single workshops for children, adolescents, and adults). However, delivery agents from two districts reflected that SIP*smart*ER was better designed to foster behavior change and could serve as a next step to that program. Future training approaches may want to address how SIP*smart*ER compares to existing programs to underscore the uniqueness and benefit in helping participants change behavior.

Our findings reflect barriers previously identified related to staff turnover, leadership, and agency structure [46–48]. Staff turnover was particularly noticeable in this D&I pilot as 75% of the districts had changes in medical directors early in the study. The medical directors were key in the promotion and support of SIP*smart*ER within their districts. This occurrence is important as past research has demonstrated that leadership is a necessary factor to further implement evidence-based programs within health departments [48–50]. Allen and colleagues’ findings suggest that effective leaders within health departments did not just talk about valuing and supporting evidence-based programs but also created an environment that fostered consistent conversation about the evidence-based programs and provided a supportive organizational environment [48]. Hu and colleagues identified that public health agencies with “high agency leadership” and “supportive workplace” were 2.08 times and 1.74 times more likely to use research evidence in the workplace compared to unsupportive environments [50]. In this trial, these changes in medical directors in the three districts may have impacted specific leadership actions related to this pilot study, which could have impacted implementation outcomes. However, researchers cannot control staff turnover, so these finding stresses the need to cultivate multiple formal and informal leaders within the health districts (e.g., opinion leaders, internal implementation leaders, and champions) from early on. In doing so, if one leader leaves the organization or the project, others remain to maintain the support and legitimacy of the program and to drive forward the implementation of the intervention [51].

Agency structures and processes influence the ability and motivation of delivery agents to complete delivery expectations and implementation strategy activities. The impact of agency structure and processes on implementation outcomes was noticeable in this trial. Notably, from interviews and capacity surveys, it was evident that in three of the four districts, delivery agents adhered to work schedules that were within normal business hours. While there was flexibility to adjust schedules and there was specific funding for the delivery agent time during the work day, they did not feel that it was appropriate, acceptable, or feasible both in terms of time and resources for them to make calls to participants during normal work hours and/or to adjust their schedules to accommodate the calls. Therefore, those agents who attempted teach-back and missed class calls did so during normal business hours. Also, as this type and scale of participant recruitment was not common practice in any of the districts, the health districts were lacking both the structure and capacity to efficiently recruit for the program.

The approach to this trial was pragmatic. Planning was guided by the RE-AIM framework with a goal to design an implementation approach that would allow SIP*smart*ER be able to significantly impact SSB intake, have a broad reach, and be readily adopted, implemented, and sustained in a typical community delivery setting, i.e., local health departments. Additional pragmatic decisions were made to allow for health departments to test out the program (i.e., trialability) [22, 28]. For example, it was decided from the outset that delivery agents would not play a role in the administration of the IVR call system and later decided they would not manage participant retention activities. These decisions were purposeful in order to allow delivery agents the ability to gain experience with recruitment, lesson delivery, and execution of teach-back and missed class calls.

Taken together, the findings and their implications identify next steps and implications for the translation of SIP*smart*ER into practice. Potential next steps include working with these four and other rural, local health districts to create systems to identify potential participants and streamline recruitment efforts. Additionally, it will be important to assess the feasibility of health department staff managing the automated call and participant retention portions of the program while also delivering the classes and teach-back and missed class calls. Finally, testing the implementation and effectiveness of the program with different combinations of components (lessons, automated calls, teach-back calls, and missed class calls) would aid in determining the most effective and feasible model.

### Limitations

Findings are limited by the small number of health districts and delivery agents included in this study and that the geographic location of the health districts are within one state health department. While this may impact generalizability, it is important to note that our mixed-methods design allowed for a robust analysis and found differences in implementation experiences and perceptions across districts. Also, the districts represent the targeted region – rural Appalachia – for future dissemination.

## Conclusions

Findings suggest SIP*smart*ER’s group classes and implementation strategy are appropriate and acceptable to health district delivery agents and can be feasibly implemented with high fidelity within the structure of rural, local public health districts in rural Virginia. However, the execution of teach-back and missed class calls as well as recruitment efforts are perceived as less appropriate and acceptable and may, therefore, be less feasible to faithfully implement. These findings, in conjunction with those related to the effectiveness of the intervention when implemented in this system, will be used to inform the further translation of SIP*smart*ER into this system.

## Additional files

Additional file 1: Post-training survey and interview guide. Data reported in Table 4 and Table 5. These measures captured delivery agents’ perceptions of the appropriateness of SIPsmartER and its components within the health district and the delivery agents’ regular job functions and their confidence to complete delivery expectations and implementation strategies and their perceived feasibility of doing so. (PDF 81 kb)

Additional file 2: Fidelity checklists. Data reported in Table 3. Fidelity checklists captured the degree to which a specific lesson’s activities were completed and if the activity was modified. (PDF 142 kb)

Additional file 3: Post-cohort survey and interview guide. Data reported in Table 4 and Table 5. These measures assessed feasibility and acceptability of SIPsmartER, its components, and the implementation strategy as well as fidelity to delivery expectations. (PDF 220 kb)

Additional file 4: Capacity Survey. Data reported in Table 5. This survey assessed the acceptability and appropriateness of maintaining SIPsmartER in their health district, including the resources they would need to sustain the program. (PDF 35 kb)

## Data Availability

The datasets used and/or analysed during the current study are available from the corresponding author on reasonable request.

## Abbreviations

IVR: Interactive Voice Response
SSB: sugar sweetened beverages
VDH: Virginia Department of Health
