# Supplementary material for: SIPsmartER delivered through rural, local health districts: adoption and implementation outcomes
Source: BMC Public Health. 2019 Sep 18;19:1273. doi: 10.1186/s12889-019-7567-6 (PMC6751747; doi:10.1186/s12889-019-7567-6)
Supplement: Supplementary file 1 — Post-training survey and interview guide. Data reported in Table 4 and Table 5. These measures captured delivery agents’ perceptions of the appropriateness of SIPsmartER and its components within the health district and the delivery agents’ regular job functions and their confidence to complete delivery expectations and implementation strategies and their perceived feasibility of doing so. (PDF 81 kb) [file 12889_2019_7567_MOESM1_ESM.pdf]

Name: ____________________________________ District: ______________________________

Thank you for participating in this survey. It will ask you to rate the training and materials as well as your confidence and perceived feasibility related to different parts of SIP*smart*ER. We will explore your responses more in an interview that will follow this survey.

(1) Please rate your satisfaction with …

	Completely Dissatisfied	Mostly Dissatisfied	A Little Dissatisfied	A Little Satisfied	Mostly Satisfied	Completely Satisfied	Comments
a) the length of the in-person training							
b) the way material was presented during the in-person training							
c) the SIPsmartER lesson plans							
d) the SIPsmartER PowerPoints							
e) the SIPsmartER participant worksheets							

(2) Please rate your confidence to complete the feasibility of completing each action

	After completing the in-person training, how confident are you that you could						Given your current role at work, how feasible is it for you to adequately prepare for each lesson					
	Extremely Unconfident	Mostly Unconfident	A Little Unconfident	A Little Confi-dent	Mostly Confi-dent	Completely Confident	Extremely Unfeasible	Mostly Unfeasible	A Little Unfeasible	A Little Feasible	Mostly Feasible	Completely Feasible
a) adequately prepare for each lesson												
	Comments:						Comments:					
b) contact participants to remind them about the upcoming classes												
	Comments:						Comments:					
c) lead Lesson 1 in a way that meets lesson objectives												
	Comments:						Comments:					
d) lead Lesson 2 in a way that meets lesson objectives												
	Comments:						Comments:					
e) lead Lesson 3 in a way that meets lesson objectives												
	Comments:						Comments:					

	After completing the in-person training, how confident are you that you could						Given your current role at work, how feasible is it for you to adequately prepare for each lesson					
	Extremely Unconfident	Mostly Unconfident	A Little Unconfident	A Little Confi-dent	Mostly Confi-dent	Completely Confident	Extremely Unfeasible	Mostly Unfeasible	A Little Unfeasible	A Little Feasible	Mostly Feasible	Completely Feasible
f) deliver the teach-back call after Lesson 1												
	**Comments:**						**Comments:**					
g) deliver the missed class calls after each lesson												
	**Comments:**						**Comments:**					
h) you could meet the learning needs of your group of participants when delivering the lessons or completing the calls												
	**Comments:**											
i) help participants learn how to accurately complete their Drink Diaries												
	**Comments:**											
j) help participants develop their own personal action plans												
	**Comments:**											
k) could facilitate discussions with participants about their barriers to drinking fewer sugary drinks and the strategies they could use to overcome barriers												
	**Comments:**											
l) answer participants' questions related to key content areas												
	**Comments:**											
m) complete fidelity check-lists after each lesson												
	**Comments:**						**Comments:**					
n) track participant attendance after each lesson/call												
	**Comments:**						**Comments:**					
o) share the necessary materials with SIPsmartER staff after each lesson/call												
	**Comments:**						**Comments:**					

	After completing the in-person training, how confident are you that you could						Given your current role at work, how feasible is it for you to adequately prepare for each lesson					
	**Extremely Unconfident**	**Mostly Unconfident**	**A Little Unconfident**	**A Little Confi-dent**	**Mostly Confi-dent**	**Completely Confident**	**Extremely Unfeasible**	**Mostly Unfeasible**	**A Little Unfeasible**	**A Little Feasible**	**Mostly Feasible**	**Completely Feasible**
p) complete lesson debriefings with SIPsmartER staff after each of the lessons												
	**Comments:**						**Comments:**					
q) get the support you need from SIPsmartER staff to deliver the program												
	**Comments:**											

OPENING

1) What makes SIPsmartER important for your health department to implement?

PERCEPTIONS OF SIPsmartER SCREENING AND RECRUITMENT

2) Please describe what you think have been the successful approaches to the current screening / recruitment process. Please be as specific as possible.

3) What do you see as unsuccessful approaches to the current screening and recruitment process?

    a. What made this unsuccessful?

    b. How do you think the process could be more successful?

    c. What other improvements would you suggest to make the screening and recruitment process training better for further health educators. Please be as specific as possible.

4) If you were leading screening and recruitment for your district (without our involvement), what would be different about your process?

SATISFACTION WITH TRAINING

5) Please describe what you think were the strengths of the in-person training. Please be as specific as possible.

6) What improvements would you suggest to make the training better for further health educators. Please be as specific as possible.

PERCEPTIONS OF THE TRAINING USEFULNESS

7) Please describe how you believe that attending the SIPsmartER training will impact you and your ability to deliver SIP*smart*ER.

PERCEPTIONS OF SIPsmartER IMPLEMENTATION

8) What sort of barriers do you expect to face as you deliver SIPsmartER?

    a. How do you anticipate addressing those barriers?

    b. What extra support do you think you will need from SIP*smart*ER staff to overcome those barriers?

9) When you think about the tasks required to implement SIP*smart*ER, what are tasks that might be difficult given for you to complete? [*Tasks may include preparing for class; delivering lessons to meet objectives, helping participants complete action plans, conducting missed class or teach-back calls, etc. Before the interview, review the participants' survey to see if any specific tasks were highlighted as not being feasible.*]

    a. What makes some tasks less feasible than others?

   b. Could any of these reasons be overcome?

      i. What might be ways to do so OR what would prevent them from being overcome?

   c. For these tasks that are not very feasible, what do you think would need to be done in the future to help health departments be able to complete these tasks.

10) What sort of existing personal skills or resources do you expect to use as you deliver SIPsmartER?

CLOSING

11) Please share any other feedback you have about the SIPsmartER training and recruitment process, including anything that earlier questions have not asked about.
