# Supplementary material for: SIPsmartER delivered through rural, local health districts: adoption and implementation outcomes
Source: BMC Public Health. 2019 Sep 18;19:1273. doi: 10.1186/s12889-019-7567-6 (PMC6751747; doi:10.1186/s12889-019-7567-6)
Supplement: Supplementary file 2 — Fidelity checklists. Data reported in Table 3. Fidelity checklists captured the degree to which a specific lesson’s activities were completed and if the activity was modified. (PDF 142 kb) [file 12889_2019_7567_MOESM2_ESM.pdf]

Date: ____ Person Completing Form: _______________________________

Site: ____ District: ____

Facilitator: ____ Start Time: ____________ End Time: ___________

(Include comments about attendance, general timeliness, classroom environment; things that went well; things that did not go well, suggestions for changes)

Use the section below to rate the level of completion of each activity: none (N); partial (P), or all (A). Also, identify if any modifications were made to activities: no (N) or yes (Y). In "Notes" include details about modifications, participant engagement, and interesting points brought up by participants.

	Completed	Modified	Notes
Teaching materials present			
Technology present and working			
**Component 1: Lesson Welcome**			
Introductions of facilitators			
Introduction of participants			
Explain purpose of SIPsmartER			
Facilitate discussion of personal reasons to reduce intake			
Review learning objectives			
**Component 2: Distinguish between sugary and non-sugary drinks**			
Review 7 categories of sugary drinks			
Drink sorting activity			
Use *What I am Drinking Worksheet?* to identify sugary drinks			
**Component 3: Review portion sizes**			
"Normal pour" activity			
Review common serving sizes			
Review 3 key ways to know a drink's sizes			
**Component 4: Calculate daily sugary drink intake**			
Use *What I am Drinking Worksheet?* to identify usual ounces			
Sugar packet counting activity & talk			
**Component 5: Recognize health impacts of sugary drinks**			
Facilitate a brief brainstorm on health risks			*(Identify what non-supported health risks were discussed)*
View the video on health impacts			
Review well-established risks			
**Component 6: Compare personal intake to recommendations**			
Communicate daily sugary drink recommendation ($\leq$ 8 oz)			
Comparison of an 8 oz cup to other common cups and containers			
Have participants compare their intake to the recommendation			

	Completed	Modified	Notes
**Component 7: Declare readiness to make a change**			
Have participants state intention to stop / cut back			
Share that SIPsmartER will support their reduction goals			
Introduce the concept and importance of the *Action Plan*			
**Component 8: Discuss reasons for reducing sugary drink intake**			
Facilitate deeper discussion about motivations			
Have participants record personal motivators on *Action Plan*.			
Encourage writing personal and specific motivations			
**Component 9: Set short and long-term goals for reducing sugary drinks**			
Have participants record their current intake on *Action Plan*			
Have participants record a short term goal for the next week			
Have participants record a long term goal (end of the program)			
Encourage realistic goals to help them get to recommendation			
**Component 10: Discuss barriers for reducing sugary drink intake**			
Facilitate a discussion of barriers (incorporating past comments and barrier/strategy cards)			
Have record 1 to 4 personal barriers on *Action Plan*			
Encourage participants to make barriers specific and personal			
**Component 11: Discuss strategies to overcome barriers**			
Facilitate group discussion on ways to overcome barriers (incorporating slides, barrier cards, and/or Tips worksheets)			
Review how can tell sugary drinks			
Conduct lemonade taste test			
Review how can tell sizes			
Review how can save money			
Review artificial sweetener evidence			
Have participants record personal strategies on their *Action Plan*			
Encourage writing personal, specific, and actionable strategies			
**Component 12: Review how to track sugary drink intake**			
Discuss the importance of tracking progress			
Show participants how to calculate averages			
**Component 13: Recap key lesson points**			
Review main			
**Component 14: Review Next Steps & Goodbye**			
Tell participants about tracking their weekly SSB intake			
Tell participants about the Teach Back call			
Tell participants about the IVR calls and their dates			
Thank participants for coming			

SIP smarter

Date:	____________________	Person Completing Form:	____________________
Location:	____________________	District:	____________________
Facilitator:	____________________	Start Time: ____________	End Time: ___________

For Facilitator

- ☐ Watch lesson framing and activity overview video
- ☐ Watch lesson background video
- ☐ Had a lesson review call with Kathleen
- ☐ Completed lesson prep in another way (describe)

Overall Impressions

(Include comments about attendance, general timeliness, classroom environment; things that went well; things that did not go well, suggestions for changes)

Use the section below to rate the level of completion of each activity: none (N); partial (P), or all (A). Also, identify if any modifications were made to activities: no (N) or yes (Y). In "Notes" include details about modifications, participant engagement, and interesting points brought up by participants.

	Completed	Modified	Notes
Teaching materials present			
Technology present and working			
**Component 1: Lesson welcome**			
Introductions of facilitators			
Introduction of participants			
Remind about purpose of SIPsmartER			
Review key points from Lesson 1			
**Component 2: Review group progress and plan for future**			
Facilitate discussion about experiences since the last class			
Share the groups' overall change in ounces of sugary drinks			
**Component 3: Today's Lesson**			
Review lesson learning objectives			
**Component 4: Discuss where and how we see advertising in our lives**			
Facilitate discussion on advertising of sugary drinks that participants see on a daily basis			
Emphasize that sugary drink advertising is very prevalent			
Emphasize that sugary drink companies are large and powerful			

	Completed	Modified	Notes
**Component 5: Discuss the main goal of advertising**			
Complete the *Media Wheel* activity, including discussion			*(Identify what non-supported health risks were discussed)*
Define seller, buyer, and product in the advertising world and apply to sugary beverage company advertising practices			
**Component 6: Explain advertising techniques**			
Explain that advertising is a form of persuasion that is effective			
Have participants complete *Name the Image* activity			
Have participants complete *Name that Jingle* activity			
Have participants complete *Name that Slogan* worksheets			
Review the *Persuasion Pie*, with examples of common marketing/advertising techniques			
**Component 7: Critique sugary drink advertisements**			
Review the main questions people should ask themselves when looking at advertisements			
Have participants verbally critique at least one television, print, radio, or packaging advertisement, using *Advertising Analysis* worksheet as appropriate			
**Component 8: Create sugary drink counter ads**			
Have participants use the persuasion techniques they learned to modify common slogans			
Have participants use the persuasion techniques they learned to create truthful sugary drink labels			
**Component 9: Recap key lesson points**			
Review four main points			
**Component 10: Create new personal action plans**			
Facilitate a discussion of barriers and strategies (incorporating past comments and barrier/strategy cards, *Making It Easier* packet)			
Walk participants through completing all parts of the *Action Plan*			
Encourage participants to make the *Action Plan* personal			
**Component 11/12: Next Steps & Goodbye**			
Encourage participants to keep tracking their SSB intake in their Drink Diaries			
Remind participants about IVR procedures and share call dates with them			
Share with participants date of the next class			
Thank participants for coming and wish them luck			

SIP smarter

Date: ______________________ Person Completing Form: ______________________

Location: ______________________ District: ______________________

Facilitator: ______________________ Start Time: __________ End Time: __________

For Facilitator

- ☐ Watch lesson framing and activity overview video
- ☐ Watch lesson background video
- ☐ Had a lesson review call with Kathleen
- ☐ Completed lesson prep in another way (describe)

Overall Impressions

(Include comments about attendance, general timeliness, classroom environment; things that went well; things that did not go well, suggestions for changes)

Use the section below to rate the level of completion of each activity: none (N); partial (P), or all (A). Also, identify if any modifications were made to activities: no (N) or yes (Y). In "Notes" include details about modifications, participant engagement, and interesting points brought up by participants.

	Completed	Modified	Notes
Teaching materials present			
Technology present and working			
**Component 1: Lesson welcome**			
Introductions of facilitators			
Introduction of participants			
Explain purpose of SIPsmartER			
Review key points from Lessons 2 and 3			
**Component 2: Review group progress and plan for future**			
Facilitate discussion about their between class experiences			
Share and celebrate group change in ounces of sugary drinks			
**Component 3: Today's Lesson**			
Review lesson learning objectives			
**Component 4: Review and practice how to read food labels**			
State how the food label is always accurate while the packaging is considered advertising, using appropriate visuals			
Review the parts of the food label important to consider when reducing sugary drinks			
Review how to calculate calories and grams per container			
Have participants complete the *Sugary Drink Comparison* handout, including reviewing answers			
Have participants use the *Drink Ranking* handouts to order the beverages from least to most sugary			
Discuss participant rankings and rationales and link answers to the Red, Yellow, and Green drinks discussed in Class 1			

SIP smarter

	Completed	Modified	Notes
**Component 5: Review the relationship of calories to weight gain and weight loss**			
Facilitate a discussion about what participants know about calories			
Illustrate the concept of calories in relationship to energy			
Illustrate energy balance			
Emphasize the energy dense (caloric) nature of sugary drinks and the ease of which we consume a lot of them			
Show the *Are You Pouring on the Pounds* video			
**Component 6:Calculate possible weight savings of reducing intake**			
Review how one can prevent weight gain by cutting back or removing sugary drinks, using Mary case study			
Have participants complete the *My Calorie Need*s worksheet			
Have participants complete the *My Calorie Saving* worksheet			
Facilitate a discussion with the group about what they learned from the activities			
**Component 7:Financial costs of drinking sugary drinks**			
Describe how too many sugary drinks can contribute to higher health care costs.			
Have participants use the *Sugary Drink Savings* worksheet (including checks)			
Facilitate a discussion on reactions to the amounts they used to spend and could save.			
**Component 8: Create sugary drink counter ads**			
Explain the purpose and design of the NGNG cards			
Have participants complete the NGNG cards			
**Component 9: Recap key lesson points**			
Review three main points			
**Component 10: Create new personal action plans**			
Facilitate a discussion of barriers and strategies using past comments and barrier/strategy cards and Tips packet)			
Have participants complete all parts of the *Action Plan*			
Encourage participants to make the *Action Plan* personal			
**Component 11/12: Next Steps & Goodbye**			
Tell participants about the IVR call			
Remind participants about IVR procedures			
Have participants sign-up for a health screening appointment			
Thank participants for coming and wish them luck			
