# Supplementary material for: SIPsmartER delivered through rural, local health districts: adoption and implementation outcomes
Source: BMC Public Health. 2019 Sep 18;19:1273. doi: 10.1186/s12889-019-7567-6 (PMC6751747; doi:10.1186/s12889-019-7567-6)
Supplement: Supplementary file 3 — Post-cohort survey and interview guide. Data reported in Table 4 and Table 5. These measures assessed feasibility and acceptability of SIPsmartER, its components, and the implementation strategy as well as fidelity to delivery expectations. (PDF 220 kb) [file 12889_2019_7567_MOESM3_ESM.pdf]

Name:________________________________ District:_____________________________

Thank you for participating in this survey. It will ask you to rate and describe your experience with different parts of SIP*smart*ER so we can learn more about what worked or did not work for you and how to improve the program and its supportive systems for the future. We will explore your responses more in an interview that will follow this survey.

My Experience Delivering of SIP*smart*ER Lessons

This first set of questions asks you to rate how true each of the statements are for you. These statements reflect what you think about different aspects of your delivery of SIP*smart*ER. There is room to explain your responses if you wish to do so.

**As the delivery agent, I felt that I was able to:**	**Very not true**	**Not true**	**True**	**Very true**	**n/a**	**Comments**
1) adequately prepare for each lesson	☐	☐	☐	☐	☐	
2) lead Lesson 1 in a way that meets lesson objectives	☐	☐	☐	☐	☐	
3) lead Lesson 2 in a way that meets lesson objectives	☐	☐	☐	☐	☐	
4) lead Lesson 3 in a way that meets lesson objectives	☐	☐	☐	☐	☐	
5) meet the learning needs of my group of participants when delivering the lessons	☐	☐	☐	☐	☐	
6) help participants learn how to accurately complete their Drink Diaries	☐	☐	☐	☐	☐	
7) help participants develop their own personal action plans	☐	☐	☐	☐	☐	
8) facilitate discussions with participants about their barriers to drinking fewer sugary drinks and the strategies they could use to overcome the barriers	☐	☐	☐	☐	☐	
9) answer participants' questions related to key content areas (e.g., what makes a sugary drink a sugary drink, media literacy, label readings).	☐	☐	☐	☐	☐	
10) get the support I needed from SIP*smart*ER staff to deliver the program	☐	☐	☐	☐	☐	

The Helpfulness of the Support Provided by the SIP*smart*ER Team

This next set of questions asks you to rate how helpfulness useful about your perceptions of the support the SIP*smart*ER team provided you during your time implementing the program. There is room to explain your responses if you wish to do so.

	Very unhelpful	Unhelpful	Helpful	Very helpful	Comments
11) In-person training	☐	☐	☐	☐	
12) Supply bins	☐	☐	☐	☐	
13) Lesson plans (*not slide sets)*	☐	☐	☐	☐	
14) Pre-lesson phone calls/meetings with Kathleen	☐	☐	☐	☐	
15) Kathleen (& Donna) attending classes	☐	☐	☐	☐	
16) Completing the fidelity check-lists	☐	☐	☐	☐	
17) Post-lesson recap meetings with Kathleen	☐	☐	☐	☐	

Thank you for participating in this interview. The purpose of this interviews is to learn about your experience delivering SIPsmartER so that we can understand more about what worked or did not work for you. Also, it will help us understand how to improve the program and its support system for the future.

My Experience Delivering of SIP*smart*ER Lessons

This first set of questions asks you about your experience delivering the SIPsmartER Lessons.

1) What were some notable positive experiences from your delivery of the SIP*smart*ER classes? *Prompt: What made these experiences positive?*

2) What were some notable negative experiences from your delivery of the SIP*smart*ER classes? *Prompt: What made these experiences negative?*

3) What were the biggest barriers your faced when delivering the SIP*smart*ER lessons? What did you do to (try to) overcome these barriers

4) What were some things you did to make the SIP*smart*ER classes “your own”? *Prompt: What were your reasons for making these decisions/modifications?*

5) If you were to deliver SIP*smart*ER classes again, what would you change?

My Perceptions of SIPsmartER Lesson Materials

For the SIPsmartER lessons, there were five types of materials: lesson plans, PowerPoint slides, worksheets, barrier cards, and tips packet. We would like to know a bit more about what you thought about each of them.

Lesson Plans: These documents provided a written overview of each lesson, including a lesson summary, materials needed, descriptions of the key parts of the lesson, and linked slides to specific parts of the lesson.

6) How did you use the lesson plans?

7) What did you like about the lesson plans?

8) How could we improve the lesson plans?

PowerPoint Slides: These slides were used to provide participants with visuals and summaries of lesson content during the actual lessons

9) What did you like about the slides

10) How could we improve these slides?

Worksheets, including Personal Action Plans and Drink Diaries: These worksheets allowed participants to apply class content. The Personal Action Plans and Drink Diaries allowed participants to set goals and plans and to track them.

11) What did you like about the worksheets?

12) How could we improve the worksheets?

Barrier Cards: These cards were used during the lessons, especially Lesson 1, to help participants complete the Barrier and Strategy portions of the personal action plans.

13) What did you like about these cards?

14) How could we improve these cards

Tips Packet: This packet was given to the participants during Lesson 1 and was meant to be a reference for them to use at home.

15) What did you like about this packet?

16) How could we improve this packet?

My Experience Implementing Teach-Back Calls and Missed Class Calls

This next set of questions asks you to about your experience implementing the teach-back call and/or the missed class calls.

17) Did you conduct a teach-back or missed class call? *If yes, prompt: which ones? If no, skip to next section.*

18) Can you describe the experience to me? *Prompts:* How did you go about completing the call? What was the experience like? Do you think you were able to get the main messages across? *Probe about the scripts if needed.*

19) Given your experience, what are you impressions about the usefulness of this/these calls? *Probe for details*

20) Given your experience, what are your impressions about the feasibility of conducting this/these call(s) for a whole cohort? *Probe for details*

21) What would you need to make them feasible?

22) If the teach-back and/or missed class call was to be modified, how could they be changed to make them more feasible for you and other health department educators?

The Helpfulness of the Support Provided by the SIP*smart*ER Team

This next set of questions asks you about your perceptions of the support the SIPsmartER team provided you during your time implementing the program.

23) In what ways did you feel supported by the SIPsmartER staff while delivering the program?

24) What do you think was/were the most important ways we supported you?

25) Where any of the supports we provided you not useful or poorly provided? *Probe: What made them not useful or poorly provided? What recommendations do you have for improving them?*

26) What other types of support would you have liked to have had provided to you? What other types of support might another health educator like?

Closing

27) Finally, please share any other feedback you have on the SIP*smart*ER program, including anything that earlier questions have not asked about.
