# Supplementary material for: SIPsmartER delivered through rural, local health districts: adoption and implementation outcomes
Source: BMC Public Health. 2019 Sep 18;19:1273. doi: 10.1186/s12889-019-7567-6 (PMC6751747; doi:10.1186/s12889-019-7567-6)
Supplement: Supplementary file 4 — Capacity Survey. Data reported in Table 5. This survey assessed the acceptability and appropriateness of maintaining SIPsmartER in their health district, including the resources they would need to sustain the program. (PDF 35 kb) [file 12889_2019_7567_MOESM4_ESM.pdf]

District ______________________

Health District Experiences and Wants Related to Implementing & Maintaining Evidence-Based Disease Prevention Programs

1. If you were to deliver SIPsmartER in the future, what would be your reasons to, your concerns about doing it, and the supports you would have for doing it?

**Reasons to deliver SIPsmartER again**	**Concerns/barriers about delivering SIPsmartER again?**
**Support you have for delivering SIPsmartER again**	**Things you would need to deliver SIPsmartER again**

District ______________________

These next questions on this survey ask about factors relevant to the health organizational capacity of your health district to implement disease prevention programming. Disease prevention programs are evidenced-based health promotion programs, like SIPsmartER, that can change individual behaviors associated with preventable health conditions, such as obesity. They may be part of normal services (e.g., WIC) but supplement usual care. Disease prevention programs can use one-on-one counseling, group classes, and/or environmental changes. They can be one or more sessions.

1. What are the primary disease prevention programs (not counting SIPsmartER) that your health district has implemented over the past 4 years?

**Program**	**Disease focus (e.g., obesity, addiction)**	**Behavior focus (e.g., smoking, drugs, diet)**	**Design (e.g., # lessons, one-on-one sessions, media campaign)**	**Who delivers?**	**Does the health district recruit for the program?**	**Is it evaluated? If yes, by who?**

District ____________________

2. What are some of the factors that your district has found to facilitate the implementation of disease prevention programs?

3. What are some of the factors that your district has found to hinder the implementation of disease prevention programs?

4. What skills, resources, and support does your health district have and need to implement and maintain disease prevention programs?

**District has**	**District needs**
